# Modeling Secondary Phenotypes Conditional on Genotypes in Case–Control Studies

**DOI:** 10.3390/stats5010014

**Published:** 2022-02-21

**Authors:** Naomi C. Brownstein, Jianwen Cai, Shad Smith, Luda Diatchenko, Gary D. Slade, Eric Bair

**Affiliations:** 1 Department of Biostatistics and Bioinformatics, Moffitt Cancer Center, Tampa, FL 33612, USA; 2 Department of Biostatistics, University of North Carolina at Chapel Hill, Chapel Hill, NC 27599-7420, USA; 3 Center for Translational Pain Medicine, Department of Anesthesiology, Duke University Medical Center, Durham, NC 27710, USA; 4 Faculty of Dental Medicine and Oral Health Sciences, McGill University, Montréal, QC H3A 0G1, Canada; 5 Department of Anesthesia, Faculty of Medicine, McGill University, Montréal, QC H3A 0G1, Canada; 6 School of Dentistry, University of North Carolina at Chapel Hill, Chapel Hill, NC 27599-7450, USA

**Keywords:** bootstrap, case–control studies, inverse-probability weighting, secondary analysis

## Abstract

Traditional case–control genetic association studies examine relationships between case–control status and one or more covariates. It is becoming increasingly common to study secondary phenotypes and their association with the original covariates. The Orofacial Pain: Prospective Evaluation and Risk Assessment (OPPERA) project, a study of temporomandibular disorders (TMD), motivates this work. Numerous measures of interest are collected at enrollment, such as the number of comorbid pain conditions from which a participant suffers. Examining the potential genetic basis of these measures is of secondary interest. Assessing these associations is statistically challenging, as participants do not form a random sample from the population of interest. Standard methods may be biased and lack coverage and power. We propose a general method for the analysis of arbitrary phenotypes utilizing inverse probability weighting and bootstrapping for standard error estimation. The method may be applied to the complicated association tests used in next-generation sequencing studies, such as analyses of haplotypes with ambiguous phase. Simulation studies show that our method performs as well as competing methods when they are applicable and yield promising results for outcome types, such as time-to-event, to which other methods may not apply. The method is applied to the OPPERA baseline case–control genetic study.

## 1. Introduction

Prospective studies are more straightforward and less prone to confounding than other study designs. However, they may require either extremely long follow-up periods or large sample sizes, and lack power. For rare diseases in particular, the sample sizes required in a prospective cohort study to have adequate statistical power to test hypotheses of interest may be prohibitively large. This can be especially problematic in genetic association studies, which may cost thousands of dollars per participant just to extract their genetic profiles. Retrospective case–control studies are more cost effective. The number of case–control studies focusing on the relationship between genetics and disease outcomes has grown astronomically in recent years.

It is well-known that when modeling the probability of case status in a case–control design, logistic regression may be used to model the primary outcome as if the study were prospective [1]. However, researchers may design studies based on one outcome and study secondary outcomes simultaneously or subsequently. Without proper care, the analysis of secondary phenotypes in case–control studies may be problematic. Standard unadjusted methods, such as linear and logistic regression, may be biased, inefficient, or lead to misleading inference. The standard method of unweighted regression on the full case–control sample and the method of adjusting for case status with an indicator variable have inflated type I error when the disease is not rare and when the disease is related to the secondary phenotype [2]. The popular practices of restricting to either cases only or controls only reduce efficiency and may be subject to bias.

This work arose in consideration with data from the Orofacial Pain: Prospective Evaluation and Risk Assessment (OPPERA) study [3,4]. The OPPERA study was primarily designed to identify risk factors for temporomandibular disorders (TMD). In addition to the cohort of initially TMD-free adults enrolled in the prospective cohort study, people with examiner-verified chronic TMD were enrolled to create an unmatched case–control study. A large number of putative risk factors were collected at enrollment [4]. Investigators sought to explain relationships between TMD and other chronic pain conditions. One putative risk factor of interest in its own right is the (ordinal) number of comorbid pain conditions a subject experiences. The genetic information collected may be predictive of comorbid conditions as well as of TMD.

Some methods have been proposed for analyzing secondary phenotypes in case–control studies. Subjects from a nested prospective case–control study may be weighted by the reciprocal of their probability of selection [5]. This stratum-weighted logistic regression method, also called inverse probability weighting (IPW), achieves the nominal type I error rate, but it can be less efficient than the standard unadjusted method or the method of adjusting for case status [2]. (Yet, in light of the fact that the method of adjusting for case status may have inflated type I error, the lower power of IPW is less alarming.) The IPW estimator [5] may merit a correction factor for the standard error. Another IPW-based method was developed for continuous outcomes [6,7] by fitting estimating equations separately to cases and controls and then combining the estimates to have minimal variance.

Likelihood-based methods have been developed [8,9] that are more powerful than IPW and can be used for both continuous and binary outcomes. However, the results may be biased and have inflated size when there is significant interaction between the genotypes and the original outcome [10,11]. Robust likelihood estimation approaches are provided for continuous outcomes using bootstrapping under the assumption that the secondary phenotype can be modeled with homoscedastic regression or the disease is rare [12], as well as under heteroscedasticity or higher prevalence [13]. Robust sandwich estimators have been proposed for the variance based on generalized estimating equations (GEE), which applies to both continuous [2,14] and binary outcomes [2]. A general conditional likelihood-based method [15] exists for outcomes that utilize standard link functions (identity, logit, and log), i.e., binary, continuous, and count outcomes.

Additional bootstrap estimates have been proposed for binary outcomes [16]. They derive non-linear equations relating the logistic regression coefficients to known sample sizes and prevalence estimates for the primary disease and secondary phenotype, calculate the unadjusted parameter estimate and standard error, resample parameter estimates from a normal distribution with that mean and standard error, refine the estimate for each replication by solving the aforementioned non-linear equations, and use bootstrapping for confidence intervals. They also adapt their bias correction method to the frequency-matched case–control study design and extend the IPW method to retrospective case–control studies using an estimate of primary disease prevalence to calculate weights. These methods have greater efficiency than the IPW method, but they assume that the secondary phenotype and covariates are binary and there is no gene–environment interaction [17].

When there is gene–environment interaction, estimation with adaptive weighting motivated by a Bayesian shrinkage estimator is recommended for when the disease is rare [10] or common [18]. Joint modeling based on Gaussian copulas for the analysis of multiple secondary phenotypes in the exponential family has a controlled type I error and is more powerful than the IPW method [11].

Existing methods may not be appropriate for all applications. First, the IPW method [5] may require an adjustment to the standard error and was originally only designed to apply to binary outcomes. Additional variable types are commonly analyzed in practice. More recent methods have only been explicitly derived and tested for continuous [2,6–9,12,13,15,19], binary [2,7–9,15,16,19], or count outcomes [7,15]. As far as we know, there are no methods that have been developed for and applied to secondary phenotypes outside the exponential family. Time-to-event outcomes, for example, may be of clinical interest, and may be present in large studies, such as OPPERA. Sequencing studies also utilize more complicated test statistics outside of the exponential family. Additionally, there may be a lack of user-friendly software for implementation.

In this paper, we propose a method for analyzing secondary phenotypes of general form in case–control genetic association studies. We advocate using the IPW method [5] for parameter estimation, but estimating the standard error via bootstrapping. This maintains the simplicity and intuitiveness of IPW and generalizes it to a wider variety of situations than previously applied, while providing a valid estimate of the standard error. Our method can handle arbitrary types of analyses, including time-to-event and non-parametric methods, as well as logistic regression and linear models, as described in the literature. Moreover, our method can be easily generalized to outcomes for which no existing method applies. We describe our methodology in detail in Section 2. Simulations are presented in Section 3. The method is applied to the OPPERA study in Section 4. We conclude with a discussion.

## 2. Proposed Method

Consider a case–control study consisting of n cases and m controls. Let $Z_{i}$, a p×1 vector, denote covariate information, $D_{i}$ denote the case–control status (1 = case, 0 = control), and $Y_{i}$ denote the secondary phenotype for i=1,…,n+m. For example, in the OPPERA study, $Z_{i}$ denotes the number of copies of the minor allele, $D_{i}$ is an indicator of whether participant i is a chronic case of TMD, and $Y_{i}$ is the ordinal number (0, 1, 2+) of comorbid pain conditions for participant i. (In general, $Y_{i}$ can take other forms, as described below.) If one were to ignore the case–control study design and consider the data as a random sample from the population, then one would use standard methodology to study the relationship between $Y={\left(Y_{1},\ldots ,Y_{n+m}\right)}^{\prime }$ and $Z={\left(Z_{1},\ldots ,Z_{n+m}\right)}^{\prime }$. The log-likelihood is denoted under the assumption of random sampling as $l\left(\theta |Y_{i},Z_{i}\right)$ where θ is a q×1 vector of model parameters of interest.

In our proposed method, parameter estimates $\left(\overset{ˆ}{\theta }\right)$ are generated using the IPW method of [5]. IPW simply weights standard analyses appropriately to account for the oversampling of cases. Specifically, in a prospective (nested) case–control study, if we denote $f_{ca}$ as the sampling fraction for cases and $f_{co}$ as the sampling fraction for controls, we use $w_{i}=1$ as the weight for cases and $w_{i}=\frac{f_{ca}}{f_{co}}$ as the weight for controls. For retrospective case–control studies, the weights may be estimated as in [16] by $w_{i}=1$ for cases and $w_{i}=\frac{n\left(1-p_{e}\right)}{mp_{e}}$ for controls, where $p_{e}$ is the estimated prevalence of cases in the population. We may write $w_{i}\left(D_{i}\right)=D_{i}+\left(1-D_{i}\right)w_{i}$.

The weighted log-likelihood is the weighted sum of the log-likelihood of each observation

(1) $$l_{W}(\theta |Y,Z,D)=\sum_{i=1}^{m+n}w_{i}\left(D_{i}\right)l\left(\theta |Y_{i},Z_{i}\right),$$

where $l\left(\theta |Y_{i},Z_{i}\right)=\text{log}\left[f\left(Y_{i}|Z_{i}\right)\right]$ if Y is continuous with pdf $f\left(Y_{i}|Z_{i}\right)$, or $l\left(\theta |Y_{i},Z_{i}\right)=\text{log}\left[P\left(Y_{i}|Z_{i}\right)\right]$ if Y is discrete with pmf $\left.P\left(Y_{i}|Z_{i}\right)\right]$.

As a simple example, suppose $Y_{i}$ is continuous, and let ${\tilde{Z}}_{i}={\left(1,Z_{i}^{\prime }\right)}^{\prime }$ add an column of ones to the covariate information in $Z_{i}$. Then, in the setting of random sampling, $Y_{i}=\alpha^{\prime }{\tilde{Z}}_{i}+\epsilon_{i},E\left(Y_{i}\right)=\alpha^{\prime }{\tilde{Z}}_{i}$, and $Var\left(Y_{i}\right)=\epsilon_{i}\sim N\left(0,\sigma^{2}\right)$ would comprise the underlying unweighted linear model with parameter $\theta =\left(\alpha ,\sigma^{2}\right)$, where α is a (p+1)×1 vector and $\sigma^{2}\geq 0$ is a non-zero constant scalar.

In the case–control setting discussed in this paper, a weighted linear model could be utilized to estimate $\theta =\left(\alpha ,\sigma^{2}\right)$ with log-likelihood according to Equation (2):

(2) $$l_{W}(\theta |Y,Z,D)=\sum_{i=1}^{m+n}\text{log}\left[f\left(Y_{i}|Z_{i},D_{i}\right)\right]=\sum_{i=1}^{m+n}w_{i}\left(D_{i}\right)\left[-\frac{\text{log}\left(2\pi \sigma^{2}\right)}{2}-\frac{{\left(Y_{i}-\alpha^{\prime }{\tilde{Z}}_{i}\right)}^{2}}{2\sigma^{2}}\right].$$

If $Y_{i}$ is binary with covariate ${\tilde{Z}}_{i}={\left(1,Z_{i}^{\prime }\right)}^{\prime }$, then under random sampling, one could use a standard logistic regression model, $\text{log}\left[\frac{P\left(Y_{i}=1\right)}{1-P\left(Y_{i}=1\right)}\right]=\alpha^{\prime }{\tilde{Z}}_{i}$, where α has length p+1. For the case–control setting, one would typically use weighted logistic regression to estimate θ=α, with

(3) $$l_{W}(\theta |Y,Z,D)=\sum_{i=1}^{m+n}\text{log}\left[P\left(Y_{i}=1|Z_{i},D_{i}\right)\right]=\sum_{i=1}^{m+n}w_{i}\left(D_{i}\right)\left\{Y_{i}\alpha^{\prime }{\tilde{Z}}_{i}-\text{log}\left[1+\text{exp}\left(\alpha^{\prime }{\tilde{Z}}_{i}\right)\right]\right\}.$$

If $Y_{i}$ is ordinal with K levels, denoted (1,2,…,K), then under random sampling, one may use a proportional odds model with cumulative logits [20] to estimate θ=(ζ,β), where $\zeta =\left(\zeta_{1},\ldots ,\zeta_{K-1}\right)$ denotes intercepts, and

(4) $$\text{log}\left[\frac{P\left(Y_{i}\leq k\right)}{1-P\left(Y_{i}\leq k\right)}\right]=\zeta_{k}+\beta^{\prime }Z_{i}=\beta^{\prime }Z_{i}+\sum_{j=1}^{K-1}\zeta_{j}I(j=k),$$

for k=1,…,K-1, leading to cumulative probabilities,

(5) $$P\left(Y_{i}\leq k|Z_{i}\right)=\frac{\text{exp}\left(\zeta_{k}+\beta^{\prime }Z_{i}\right)}{1+\text{exp}\left(\zeta_{k}+\beta^{\prime }Z_{i}\right)}=\frac{\text{exp}\left[\beta^{\prime }Z_{i}+\sum_{j=1}^{K-1}\zeta_{j}I(j=k)\right]}{1+\text{exp}\left[\beta^{\prime }Z_{i}+\sum_{j=1}^{K-1}\zeta_{j}I(j=k)\right]}.$$

The cumulative probabilities from (5) may be used to calculate individual probabilities into the likelihood, where

$$P\left(Y_{i}=1|Z_{i}\right)=P\left(Y_{i}\leq 1|Z_{i}\right),\;P\left(Y_{i}=k|Z_{i}\right)=P\left(Y_{i}\leq k|Z_{i}\right)-P\left(Y_{i}\leq k-1|Z_{i}\right)\;\text{for}\;k=2,\ldots ,K-1,\;\text{and}\;P\left(Y_{i}=K|Z_{i}\right)=1-\sum_{k=1}^{K-1}P\left(Y_{i}=k|Z_{i}\right).$$

Then the weighted likelihood under case–control sampling is:

(6) $$l_{W}(\theta |Y,Z,D)=\sum_{i=1}^{m+n}\text{log}\left[P\left(Y_{i}|Z_{i},D_{i}\right)\right]=\sum_{i=1}^{m+n}w_{i}\left(D_{i}\right)\;\text{log}\left[P\left(Y_{i}=k|Z_{i}\right)\right].$$

If ($Y_{i},\Delta_{i}$) is a (possibly censored) time-to-event outcome with failure time $T_{i}$ and censoring time $C_{i},Y_{i}=\text{min}\left(T_{i},C_{i}\right)$ and $\Delta_{i}=I\left(T_{i}<C_{i}\right)$, then one may use a Cox proportional hazards model [21] for the estimation of θ=β

(7) $$\lambda_{T_{i}}\left(t|Z_{i}\right)=\lambda_{0}(t)\;\text{exp}\left(\beta^{\prime }Z_{i}\right)$$

with weighted log-partial-likelihood given by

(8) $$l_{W}(Y|Z,D)=\sum_{i=1}^{m+n}w_{i}\left(D_{i}\right)\Delta_{i}\left\{\beta^{\prime }Z_{i}-\text{log}\left[\sum_{l=1}^{m+n}w_{l}\left(D_{l}\right)I\left(Y_{i}<Y_{l}\right)\text{exp}\left(\beta^{\prime }Z_{l}\right)\right]\right\}.$$

We propose the use of bootstrapping to estimate the standard error of the estimate of interest, $\text{se}(\overset{ˆ}{\theta })$, in any of the aforementioned scenarios. We select R samples from the empirical distribution of the original data. For each bootstrap replication, we apply the IPW method described above. Specifically, let (D,Y,Z) denote the data with empirical cdf f, and let $\left(D_{r}^{*},Y_{r}^{*},Z_{r}^{*}\right)$ denote bootstrap replications for r=1,…,R. The first step is to fit a model to $\left(D_{r}^{*},Y_{r}^{*},Z_{r}^{*}\right)$ using the weighted log likelihood (1) for each replication. The variance of the parameter estimate $\overset{ˆ}{\theta }$ is given by the estimated variance of the R bootstrap parameter estimates of θ. Confidence intervals may be generated by the percentile method, bias-corrected and accelerated (BCa) method, approximate bootstrap confidence interval (ABC) method, bootstrap-t, or a normal approximation [22,23]. Standard software, such as the boot package in R [24,25], can easily generate these estimates.

## 3. Simulation Study

### 3.1. General Setup

We simulated data under the framework in [8]. In order to have n cases and m controls, we generated $n_{s}=\frac{3\;\text{max}(m,n)}{p_{e}}$ observations. This ensured there would be enough cases and controls in each dataset. For all subjects in this superset, $i=1,\ldots ,n_{s}$, we assume that the relationship between case–control status, $D_{i}$, the number of copies of the minor allele, $Z_{i}$, and the secondary phenotype, $Y_{i}$, is given by a logistic regression model based on the genetic profile and secondary phenotype. The distribution of each type of outcome and corresponding specific form of the logistic regression model are given in Sections 3.2–3.4 for binary, ordinal, and time-to-event outcomes, respectively. Each equation specifies that the probability of being a case of the primary disease (rather than a control) depends on the status of the genetic profile and the secondary phenotype. The inheritance model is assumed to be additive. We assumed a minor allele frequency of 30% and an estimated prevalence of D of 10% to approximate the 8.6% prevalence of TMD in the OPPERA prospective cohort study [26].

Finally, for each simulation run, we extracted the first n cases and m controls from the superset of $n_{s}$ to comprise the simulated case–control dataset of size m+n. For each method and scenario, we estimated the value of the parameter relating to the secondary phenotype and the number of copies of the minor allele. (This was the log-odds ratio or log-hazard ratio depending on the type of outcome.) Average bias, empirical coverage, and average confidence interval width were compared between our method, and the naive methods that restrict to cases, restrict to controls, or adjust for case–control status using an indicator variable. We also compared the performance of the IPW with the GEE method of [2] when applicable, i.e., for continuous outcomes. All simulation types included 1000 datasets each with n=1000 cases and m=1000 controls. In each case, the average bias was estimated using the difference between the mean of the 1000 parameter estimates and the true parameter value. Analogously, the average confidence interval width produced by each method is equal to the average difference between the upper and lower confidence limits among the 1000 scenarios. Similarly, we calculated the empirical coverage probability produced by each method using the proportion of the 1000 simulations for which the true parameter value is contained in the confidence interval.

### 3.2. Continuous Phenotypes

For continuous secondary phenotypes, we assume a standard linear model with normally distributed errors, $\epsilon_{i}\sim N\left(0,\sigma^{2}\right)$,

(9) $$Y_{i}=\beta_{0}+\beta_{1}Z_{i}+\epsilon_{i},$$

where $Z_{i}$ is defined in Section 3.1, and case status is defined by

(10) $$\text{logit}\left[P\left(D_{i}=1|Y_{i},Z_{i}\right)\right]=\gamma_{0}+\gamma_{1}Z_{i}+\gamma_{2}Y_{i}.$$

Our simulations included the parameters $\beta_{0}=0,\beta_{1}=-0.12,-0.5,-1,-2$ and $\gamma_{1}=\text{log}(2),\text{log}(3),\text{log}(5),\text{log}(10),\gamma_{2}=\text{log}(2)$ and $\sigma^{2}=1$.

In order to keep the prevalence approximately constant, we set the value of $\gamma_{0}$ separately for each simulation, according to

(11) $$\overset{ˆ}{\gamma_{0}}=\text{log}\left[\frac{p_{e}\;\text{exp}(-\tilde{X})}{1-p_{e}}\right],$$

where

(12) $$\tilde{X}=\gamma_{1}\overset{‾}{Z}+\gamma_{2}\overset{‾}{Y},$$

$p_{e}=0.1$, and $\overset{‾}{Z}=\frac{1}{n_{s}}\sum_{i=1}^{n_{\text{s}}}Z_{i}$ and $\overset{‾}{Y}=\frac{1}{n_{s}}\sum_{i=1}^{n_{\text{s}}}Y_{i}$ are the averages of the $i=1,\ldots ,n_{s}.Z_{i}$ and $Y_{i}$ values.

The parameter of interest was $\beta_{1}$. Considering continuous outcomes facilitated the comparison of the IPW with the GEE method of [2].

Simulations with continuous outcomes yielded the following results. In all scenarios, our method had negligible bias and coverage rate near 95%, as desired. Performance in terms of bias, coverage, and confidence interval width was comparable to that of [2]. The bootstrapping IPW method had comparable bias to the method of [2] and less bias than all other methods. Details are found in Table 1. This shows that when competing methods are applicable, our method does at least as well as, if not better than the competitors.

### 3.3. Ordinal Phenotypes

We tested four scenarios for ordinal phenotypes with 3 levels. For simplicity, we will denote these as $Y_{i}=0,Y_{i}=1$ and $Y_{i}=2$. In general, we generated the ordinal outcomes with the following probabilities

$$p_{0}=P\left(Y_{i}=0\right)=\text{exp}\left(\zeta_{0}+\beta Z_{i}\right)/\left(1+\text{exp}\left(\zeta_{0}+\beta Z_{i}\right)\right),\;p_{1}=P\left(Y_{i}=1\right)=\text{exp}\left(\zeta_{1}+\beta Z_{i}\right)/\left(1+\text{exp}\left(\zeta_{1}+\beta Z_{i}\right)\right)-p_{0},\;p_{2}=P\left(Y_{i}=2\right)=1-p_{1}-p_{0}.$$

For all subjects, i=1,…,m+n, we assume that the relationship between case–control status, $D_{i}$, the number of minor allele copies, $Z_{i}$, and the secondary phenotype, $Y_{i}$, is given by the following logistic regression model

(13) $$\text{logit}\left[P\left(D_{i}=1|Y_{i},Z_{i}\right)\right]=\gamma_{0}+\gamma_{1}Z_{i}+\gamma_{2a}I\left(Y_{i}=1\right)+\gamma_{2b}I\left(Y_{i}=2\right),$$

where

(14) $$\overset{‾}{Y}=\gamma_{2a}\left(\frac{\sum_{i=1}^{m+n}I\left(Y_{i}=1\right)}{m+n}\right)+\gamma_{2b}\left(\frac{\sum_{i=1}^{m+n}I\left(Y_{i}=2\right)}{m+n}\right)$$

is used to define the average outcome in Equation (12) and thus the value of $\gamma_{0}$ in (11).

For the four scenarios, we used the following parameters:

1. $\beta =0.5,\zeta_{0}=1.5,\zeta_{1}=2.5$, and $\gamma_{1}=\gamma_{2a}=\gamma_{2b}=\text{log}(2)$
2. $\beta =1,\zeta_{0}=0,\zeta_{1}=1$, and $\gamma_{1}=\gamma_{2a}=\gamma_{2b}=\text{log}(2)$
3. $\beta =0.75,\zeta_{0}=1,\zeta_{1}=2$, and $\gamma_{1}=\gamma_{2a}=\gamma_{2b}=\text{log}(2)$
4. $\beta =0.5,\zeta_{0}=1.5,\zeta_{1}=2.5,\gamma_{1}=\gamma_{2a}=\text{log}(2)$, and $\gamma_{2b}=\text{log}(3)$.

Our weighted bootstrap method has less bias than all other methods. Using controls only yielded close but lower coverage in scenarios 1, 2, and 4, and identical coverage in scenario 3. However, this finding may be an artifact of the relatively low simulated prevalence of cases and may not be replicated for higher population case rates. None of the other methods have adequate coverage for these ordinal simulations. Results are given in Table 2.

### 3.4. Time-to-Event Phenotypes

Survival outcomes were generated as in [27] with exponential failure and censoring times. The failure time $T_{i}$ satisfies Equation (7) where $\lambda_{0}(t)=1$ for all t,β=-1 and $Z_{i}$ is the number of copies of the minor allele. The censoring time was exponential with shape parameter 2. The parameter of interest was β and the outcome of interest was $\left(Y_{i},\Delta_{i}\right)$ where $Y_{i}=\text{min}\left(T_{i},C_{i}\right)$ and $\Delta_{i}=I\left(T_{i}<C_{i}\right)$. This yielded about 84% censoring. High censoring was used in simulations to parallel findings in the application of interest, the OPPERA study [26].

The case status was similar to Equation (10) for continuous outcomes, but instead depended on the true failure time rather than the observed time as follows

(15) $$\text{logit}\left[P\left(D_{i}=1|Z_{i},T_{i}\right)\right]=\gamma_{0}+\gamma_{1}Z_{i}+\gamma_{2}T_{i}.$$

The value of $\gamma_{0}$ was set by Equation (11) with $\tilde{X}$ defined by Equation (12) and $\overset{‾}{X}$ and $\overset{‾}{Y}$ defined as in Section 3.2. We used $\gamma_{1}=\gamma_{2}=\text{log}(2)$.

For time-to-event outcomes, our method retained empirical coverage around 95% and had less bias than all other methods. None of the other methods have adequate coverage, except the method that adjusts for case status. However, the latter method was overly conservative. See Table 3 for details. Other methods do not apply for this type of outcome. Consequently, no comparison was made.

## 4. Data Application

We applied the method to the baseline case–control genetic study within OPPERA. The prospective cohort study consisted of 3263 healthy TMD-free volunteers and 186 volunteers determined at baseline to have TMD. All 186 cases were retained and 1633 controls were randomly selected for the baseline case–control study.

The covariates of interest were 2924 SNPs collected in a genetic association study of 3037 participants [28,29]. The outcome was the number of co-morbid conditions, categorized as either zero, one, or more than one co-morbid condition. Upon enrollment in OPPERA, participants self-reported by checking experience with a list of 20 conditions on the Comprehensive Pain and Symptom Questionnaire (CPSQ). Examples of chronic pain conditions include arthritis, fibromyalgia, irritable bowel syndrome, include chronic pelvic pain, among others. All cases and 1626 controls filled out the CPSQ, [30]. Combining these yielded 166 cases and 1435 controls with information available on both their history of comorbid conditions and their genetic profiles. Recruited from 4 study sites and ranging from 18 to 44 years in age, these 1601 individuals comprise the proceeding analysis. For more details of the OPPERA study design, see [3,4,28,30].

For each SNP with less than 5% missing values, we fit a proportional odds model to the data, adjusting for study site, age, gender, and two racial eigenvectors calculated as described in [29].

We collected the p-values and created Q–Q plots of the negative logarithm of the p-values for the standard unweighted method and for our weighted bootstrapping method. The plots indicate that neither method found any SNPs that were significantly associated with comorbid pain conditions after adjusting for multiple comparisons. See Figures 1 and 2.

## 5. Discussion

Our proposed method for the analysis of intermediate phenotypes in case–control studies of genetic data is simple and easily implemented in standard software. The simulation results indicate that it is approximately unbiased, and has comparable coverage and confidence interval width to the method of IPW with GEE [2]. Under situations in which the retrospective likelihood-based method [8] is applicable, their method should be more powerful than our proposed method.

Our method is general enough to allow for the analysis of multiple outcomes simultaneously and of outcomes for which previous methodology may not be applicable. This generality allowed the analysis of the phenotype of interest from OPPERA applied in this manuscript, namely the number of comorbid pain conditions. Although two prior methods mention potential extensions to count data [7,15], such extensions have not been explicitly demonstrated, and thus their relative performance remains unknown. Currently, our method is the only known viable way to evaluate secondary time-to-event outcomes in case–control studies. In addition, multiple outcomes could be analyzed using standard multivariate methods but weighting each observation as described in this paper, and bootstrapping to estimate the standard error. More importantly, the method can be applied to complicated test statistics where there is no existing formula for the standard error, such as the many test statistics employed in sequencing studies. We expect the methods to perform well for different case to control ratios when the sample sizes for cases and controls are sufficient. We also expect the methods to perform well with multiple but a relatively small number of markers compared with the sample size when sample sizes for cases and controls are sufficient. In situations where the number of genetic markers exceeds the sample size, our methods may not be applicable. It is worth noting that our procedure is computationally non-trivial due to the use of bootstrapping, but the runtime is reasonable for modern computers. For 1000 runs of the survival scenario with 100 bootstrap replications, for instance, the output of proc.time was 773.288, or about 13 minutes of total elapsed time, with an average of 0.77 seconds per run using 100 bootstrap replications.

## Figures and Tables

**Figure 1.**
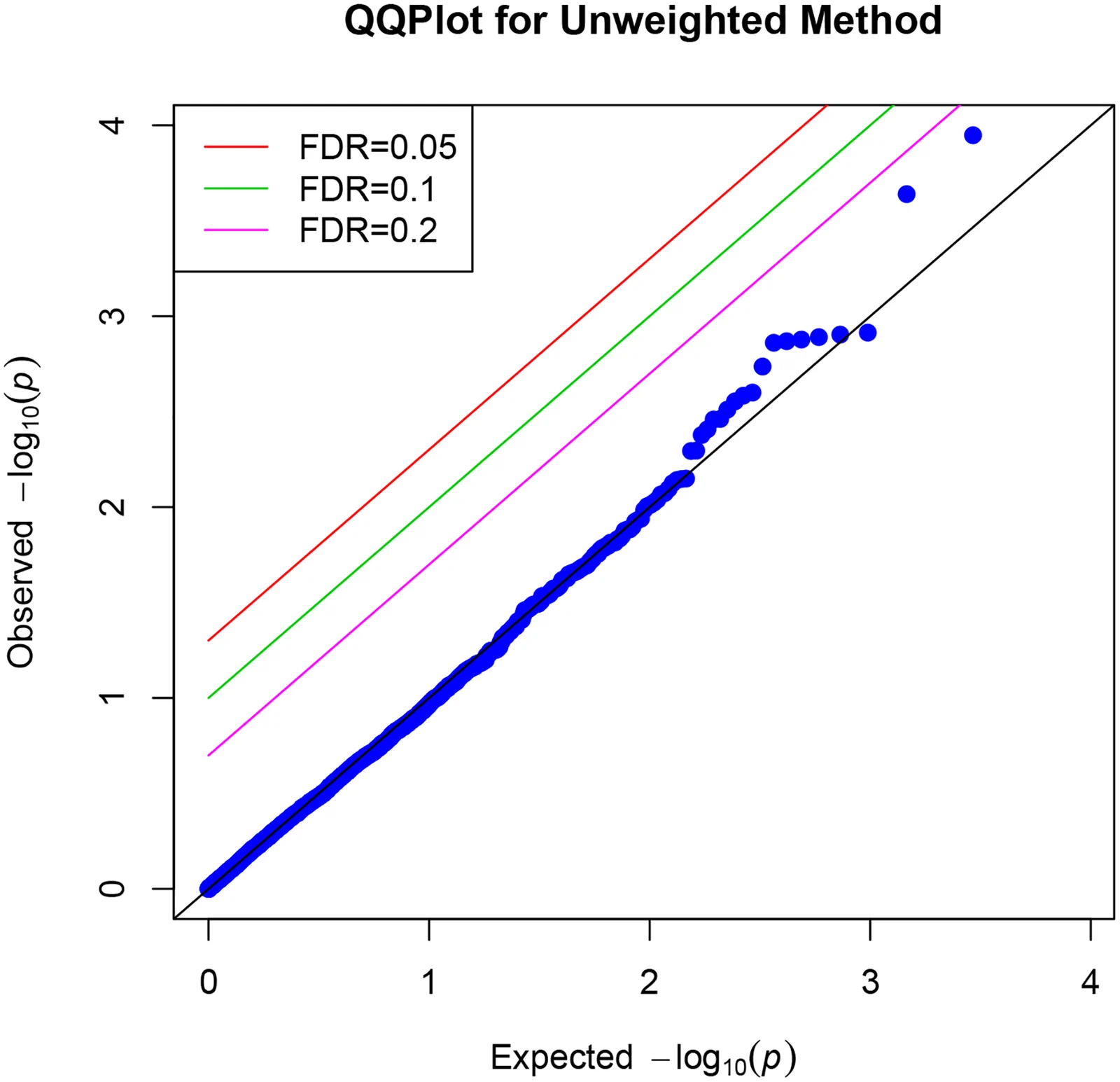
Q–Q plot for the unweighted method.

**Figure 2.**
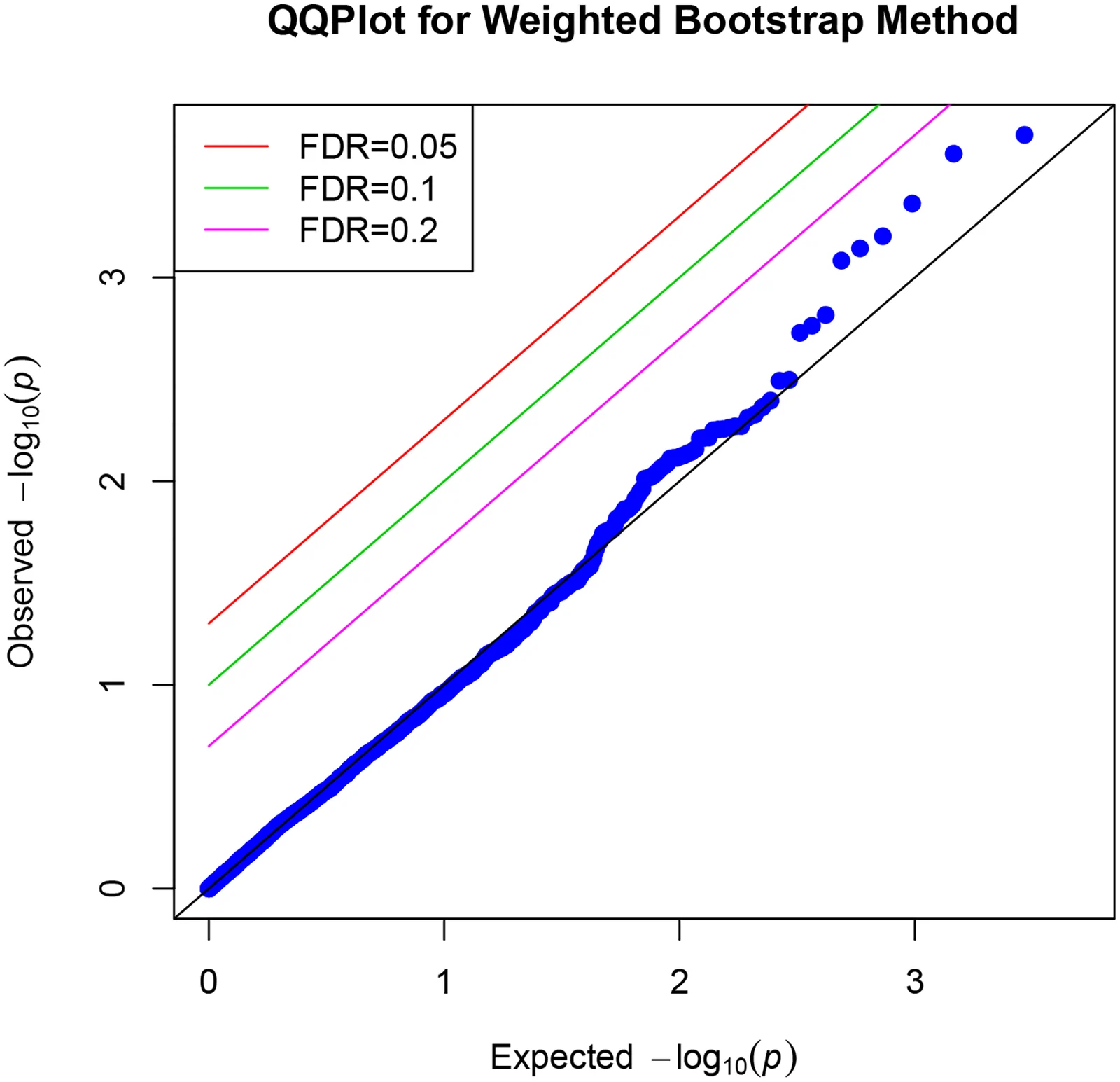
Q–Q plot for the weighted bootstrap method.

**Table 1.** Results of simulations for intermediate continuous phenotypes.

Parameters	$\beta_{1}=-0.12$ $\gamma_{1}=log(2)$		$\beta_{1}=-0.12$ $\gamma_{1}=log(3)$		$\beta_{1}=-0.12$ $\gamma_{1}=log(5)$		$\beta_{1}=-0.12$ $\gamma_{1}=log(10)$		$\beta_{1}=-0.5$ $\gamma_{1}=log(2)$		$\beta_{1}=-1$ $\gamma_{1}=log(2)$		$\beta_{1}=-2$ $\gamma_{1}=log(2)$	
	Bias	Cover	Bias	Cover	Bias	Cover	Bias	Cover	Bias	Cover	Bias	Cover	Bias	Cover
LM	−0.044	0.728	0.059	0.577	0.061	0.532	0.048	0.666	0.028	0.883	0	0.944	−0.058	0.643
LM, controls only	−0.039	0.875	−0.073	0.698	−0.108	0.444	−0.151	0.206	−0.022	0.926	−0.002	0.939	0.037	0.892
LM, cases only	−0.056	0.761	−0.099	0.394	−0.166	0.06	−0.256	0	−0.028	0.911	0.002	0.949	0.061	0.794
LM adjusted for case status	0.048	0.696	−0.088	0.235	−0.14	0.021	−0.208	0	−0.025	0.877	0	0.945	0.047	0.736
Monsees	−0.002	0.950	−0.001	0.951	0.001	0.948	0.001	0.949	0.001	0.952	−0.001	0.938	−0.003	0.949
Bootstrap	−0.002	0.950	−0.001	0.956	0.001	0.944	0.001	0.946	0.001	0.950	−0.001	0.937	−0.003	0.944
CI Width (Valid Methods Only)														
Monsees	0.164		0.160		0.155		0.148		0.166		0.167		0.168	
Bootstrap	0.162		0.160		0.154		0.147		0.165		0.167		0.168	

LM = Linear Model.

**Table 2.** Results of simulations for intermediate ordinal phenotypes.

Method	Result							
	Scenario 1		Scenario 2		Scenario 3		Scenario 4	
	Bias	Coverage	Bias	Coverage	Bias	Coverage	Bias	Coverage
Naive	−0.054	0.909	−0.068	0.836	−0.053	0.910	−0.069	0.871
Controls only	0.075	0.937	0.051	0.937	0.063	0.948	0.093	0.933
Cases only	0.805	0	−0.815	0	0.226	0	0.903	0
Adjusted for case status	0.057	0.904	0.027	0.937	0.054	0.913	0.101	0.824
Bootstrap	0.020	0.943	0.006	0.944	0.015	0.948	0.015	0.951
CI Width (Valid Methods Only)								
Bootstrap	0.519		0.399		0.477		0.512	

**Table 3.** Results of simulations for intermediate time-to-event phenotypes.

Method	Bias	Coverage
Naive	−0.457	0.006
Controls only	0.272	0.396
Cases only	−0.800	0.225
Adjusted for case status	0.091	1.000
Bootstrap	−0.017	0.944
	CI Width (Valid Methods Only)	
Bootstrap	0.439	

## Data Availability

The data and code that support the findings of this study are available from the corresponding author upon reasonable request.

## Abbreviations

The following abbreviations are used in this manuscript:

OPPERA: Orofacial Pain: Prospective Evaluation and Risk Assessment
TMD: Temporomandibular disorders
IPW: Inverse probability weighting
Q–Q: Quantile–quantile
